# Predictive value of modified systemic inflammation score for postoperative unplanned ICU admission in patients with NSCLC

**DOI:** 10.3389/fsurg.2022.893555

**Published:** 2022-08-03

**Authors:** Zhulin Wang, Hua Zhang, Chunyao Huang, Kaiyuan Li, Wenqing Luo, Guoqing Zhang, Xiangnan Li

**Affiliations:** ^1^ Department of Thoracic Surgery, First Affiliated Hospital of Zhengzhou University, Zhengzhou China; ^2^ Department of Cardiovascular surgery, Henan Provincial Chest Hospital, Zhengzhou China

**Keywords:** NSCLC, mSIS, lung cancer surgery, unplanned ICU admission, postoperative complications

## Abstract

**Background:**

The purpose of this study was to investigate the predictive value of the modified systemic inflammation score (mSIS) in postoperative unplanned admission to the intensive care unit (ICU) in patients with non-small-cell lung cancer (NSCLC).

**Methods:**

The clinical data of 1,321 patients with NSCLC treated with thoracic surgery in our hospital from August 2019 to June 2021 were analyzed retrospectively. The preoperative mSIS, which takes into account the serum albumin (ALB) level and lymphocyte-to-monocyte ratio (LMR), was recorded as 0, 1 or 2 and then was used to identify high-risk patients with unplanned admission to the ICU. The independent risk factors for unplanned admission to the ICU in patients with NSCLC after surgery were identified by multivariate logistic regression analysis.

**Results:**

A total of 1,321 patients, including 549 (41.6%) males and 772 (58.4%) females, were included. The median age was 57 years (range 16–95 years). The incidence of unplanned admission to the ICU in patients with mSIS = 2 was significantly higher than that in those with mSIS = 0 and mSIS = 1. The multivariate analysis showed that an mSIS of 2 (OR = 3.728; *P* = 0.004; 95% CI, 1.520–9.143), an alcohol consumption history (OR = 2.791, *P* = 0.011; 95% CI, 1.262–6.171), intraoperative infusion volume (OR = 1.001, *P* = 0.021; 95% CI, 1.000–1.001) and preoperative underlying diseases (OR = 3. 57, *P* = 0.004; 95% CI, 1.497–8.552) were independent risk factors for unplanned admission to the ICU after lung cancer surgery. In addition, the multivariate logistic regression model showed that the C-statistic value was 0.799 (95% CI: 0.726∼0.872, *P* < 0.001).

**Conclusions:**

The mSIS scoring system can be used as a simplified and effective predictive tool for unplanned ICU admission in patients with NSCLC.

## Introduction

Lung cancer is one of the most common malignancies. In recent years, the morbidity of lung cancer has increased significantly (1). The common methods for the treatment of lung cancer include surgery, chemotherapy and radiotherapy (1). Clinically, surgery is the most effective treatment for patients with early-stage lung cancer. With continual advances in minimally invasive thoracic surgery techniques, video-assisted thoracoscopic surgery (VATS) has become the standard operative approach for treating early-stage lung cancer (2). Numerous clinical studies have shown that video-assisted thoracoscopic surgery has a significantly lower incidence of postoperative complications than open thoracotomy (3, 4). However, postoperative complications in patients with lung cancer still occur sporadically. The risk of reoperation and poor prognosis is significantly increased when serious complications occur. According to the Clavien-Dindo complication grading system, the complications that need to be treated with intensive care in the intensive care units (ICU) are grade IV complications (5). Therefore, patients need to be admitted to the ICU to manage severe complications and obtain enhanced life support. Patients admitted to the ICU are in critical condition and have a poor prognosis and an increased risk of short-term death, which leads to the prolongation of postoperative hospital stay and imposes a substantial economic burden (6). The aims of this study were to identify patients at high risk of unplanned ICU admission by exploring the influencing factors of unplanned ICU admission and to intervene in advance to reduce the proportion of patients with unplanned ICU admission.

The modified systemic inflammation score (mSIS) is a scoring system that considers a preoperative patient's albumin level and lymphocyte to monocyte ratio (LMR) to predict prognosis. To date, the mSIS has been applied to predict the prognoses of esophageal cancer (7) and gastric cancer (8). However, there is no report on the prediction of unplanned admission to the ICU among patients with non-small-cell lung cancer (NSCLC) by the mSIS. In this study, the clinical data of 1,321 patients with NSCLC treated by thoracic surgery in our hospital from August 2019 to June 2021 were retrospectively analyzed to explore the predictive value of the mSIS in postoperative unplanned admission to the ICU.

## Materials and methods

### Patients

This study was approved by the Ethics Committee of the first affiliated Hospital of Zhengzhou University (ID:2022-KY-0325). A total of 1,321 patients with NSCLC treated by thoracic surgery in our hospital from August 2019 to June 2021 were included. Demographic information including sex, age, height, weight and other basic information was collected. Preoperative basic diseases, preoperative laboratory indicators, surgical procedures, postoperative pathological examination results and other clinical information were analyzed. All patients were evaluated for cardiopulmonary function (forced expiratory volume in one second (FEV1), forced vital capacity (FVC), ejection fraction (EF)) preoperatively (Table 1). In some previous studies (9–12), it has been found that a predicted postoperative FEV1 (ppoFEV1) of less than 0.8 L is significantly associated with an increased risk of surgical death and an increase in postoperative complications. The formula (13) for predicting postoperative FEV1 is: ppoFEV1 = preoperative FEV1 × (1-y/19) (where y is the number of segments to be removed, and 19 is the total number of segments). Therefore, ppoFEV1 = 0.8 L was used as the cutoff value of surgery in our study. A cardiopulmonary exercise test (CPET) was performed in patients with a ppoFEV1 < 0.8 L, and patients with a peak oxygen uptake (VO2max) lower than 15 ml/kg/min were excluded from the operation (14). Patients who were determined to be unsuitable for surgery after undergoing an evaluation by surgeons and anesthesiologists were given radiotherapy, chemotherapy, targeted therapy and so on. Thoracoscopy was the first choice for surgical patients. In addition, all patients underwent CT reexamination at the outpatient clinic 30 d after operation, and inpatient reexamination and treatment were performed for patients suspected of developing complications.

**Table 1:** Basic information of patients.

Characteristic	No complications (*N* = 966)	Secondary outcome (*N* = 326)	Major outcome (ICU patients, *N* = 29)	*P*-value
Age (years)	57(50, 65)	57(51, 64)	66(60.5, 71.5)	<0.001
Sex				0.003
Male	391	137	21	
Female	575	189	8	
Smoking	242	82	14	0.018
Drinking	147	55	12	0.001
mSIS				<0.001
0	587	218	14	
1	296	85	6	
2	83	23	9	
Preoperative underlying diseases				0.003
No	541	182	7	
Yes	425	144	22	
Extent of resection				0.073
Wedge resection	107	48	5	
Segmentectomy	136	55	2	
Lobectomy	704	222	22	
Other	19	1	0	
Postoperative cardiac complications				<0.001
No	934	324	17	
Yes	32	2	12	
Intraoperative infusion volume	1900(1500, 2200)	2000(1500, 2300)	2300(1550, 2800)	0.002
Operation time	135(110, 180)	140(105, 180)	170(135, 227)	0.044
EF	64(63, 65)	64(62, 65)	64(63, 65)	0.903
FVC	3.30(2.84, 3.90)	3.32(2.90, 4.00)	3.46(2.78, 3.77)	0.122
FEV1	2.54(2.17, 2.95)	2.56(2.23, 3.02)	2.47(2.03, 2.64)	0.106
FEV1%pred	95.00(87.90, 100.00)	95.00(89.08, 99.85)	91.00(76.90, 95.85)	0.116
LMR	4.53(3.55, 5.85)	4.66(3.55, 5.85)	3.59(2.87, 5.24)	0.260
NLR	1.86(1.42, 2.54)	1.82(1.39, 2.33)	2.22(1.54, 3.29)	0.745
PLR	126.38(99.05, 162.29)	122.84(100.00, 155.09)	124.64(99.04, 174.65)	0.112
PNI	50.70(47.60, 54.01)	51.12(48.00, 54.60)	50.15(44.47, 53.18)	0.276
Chest tube time	4(4, 5)	4(4, 5)	6(4, 10.5)	<0.001
Hospitalization time	13 (10, 19)	13 (10, 20)	23(15, 28.5)	<0.001

mSIS, modified systemic inflammation score; EF, ejection fraction; FVC, forced vital capacity; FEV1, forced expiratory volume in one second; FEV1%pred, forced expiratory volume in one second as a percentage of predicted value; LMR, lymphocyte-to-monocyte ratio; NLR, neutrophil lymphocyte ratio; PLR, platelet lymphocyte ratio; PNI, prognostic nutritional index.

### ICU admission definitions and criteria

Planned ICU admission was defined as a patient with lung cancer who was planned before surgery to be transferred to the ICU postoperatively. Unplanned admission to the ICU was defined as the transfer of a lung cancer patient to the ICU after returning to the general ward postoperatively, which was not planned preoperatively. ICU admission criteria: acute respiratory failure, shock, severe heart rhythm disorders, severe infection, and severe water electrolyte disorders with various critical acute reversible illnesses that could not be handled in the general ward based on the joint judgment of surgeons and senior physicians in the ICU.

### Patient selection

The inclusion criteria were as follows: 1. NSCLC as the postoperative pathological type. The exclusion criteria were as follows: 1. lung cancer without surgical treatment; 2. not NSCLC; or 3. More than 30% of patients had missing medical record data.

### Definition of preoperative underlying disease and postoperative complications

Preoperative underlying diseases were defined as diseases that already existed before admission (including cerebrovascular disease, endocrine system disease, respiratory system disease, nervous system disease and other diseases). Postoperative unplanned admission to the ICU was defined as admission to the ICU for postoperative intensive care or due to complications that required ICU care. Postoperative complications were defined and reasons for unplanned stay in the ICU were categorized as follows: (1) respiratory failure, severe pulmonary impairment requiring endotracheal intubation; (2) cerebral infarction, such as necrosis of ischemic and hypoxic lesions of brain tissue and corresponding clinical manifestations of neurological deficit due to various causes; (3) cerebral hemorrhage, such as hemorrhage caused by the rupture of blood vessels in the non-traumatic brain parenchyma, resulting in neurological dysfunction; (4) acute heart failure, as indicated by acute cardiac function decline needing emergency treatment; (5) severe renal failure, in which kidney function was seriously compromised and dialysis was needed; (6) arrhythmia of a life-threatening nature and needing urgent treatment; (7) pulmonary embolism, including massive embolism causing circulatory dysfunction that could be life-threatening.

### Definition of the mSIS

The mSIS was defined based on the combination of the preoperative serum albumin and the lymphocyte to monocyte ratio (LMR) and was scored as follows: (a) ALB ≥ 40 g/L and LMR ≥ 3.4 was assigned a score of 0, (b) either ALB < 40 g/L or a LMR < 3.4 was assigned a score of 1, and (c) ALB < 40 g/L and a LMR < 3.4 was assigned a score of 2.

### Outcomes of interest

Our outcome of interest was any Clavien-Dindo grade ≥II complication developed within 30 d after surgery. The primary outcome was Clavien-Dindo grade IV complications requiring intensive care or ICU management; secondary outcomes were Clavien-Dindo grade II-III complications, including pneumonia (fever >38°C, purulent sputum, abnormal findings on radiography) requiring antibacterial drugs, mild atelectasis, mild pneumothorax, pleural effusion requiring suction and drainage only, requirement of endoscopic intervention for complications such as severe atelectasis, severe subcutaneous emphysema and pneumothorax requiring reoperation. In-hospital mortality was defined as any death during hospitalization.

### Statistical analysis

We used IBM SPSS 22.0 software (IBM SPSS Statistics, Version 22.0; IBM Corp., Armonk, NY, USA) for data processing and statistical analysis. The chi-square test or Fisher's exact probability method was used to compare count data between groups. In the univariate analysis, variables meeting the criterion *P* < 0.20 were entered into the multivariate logistic regression model. Multivariate logistic regression analysis was used to determine the independent risk factors for unplanned admission to the ICU after lung cancer operation. Receiver operating characteristic (ROC) curves were used to evaluate the discrimination ability of the model to predict the unplanned ICU admission of patients with NSCLC.

## Results

### Patient characteristics

A total of 1,321 patients were included, including 549 (41.6%) males and 772 (58.4%) females. The median age was 57 years (range 16–95 years). Table 1 shows the clinical characteristics of the three groups of patients and the differences in preoperative cardiopulmonary function and biochemical indicators. In our study, lobectomy was performed in 948 (71.7%) patients, and preoperative underlying diseases were present in 591 (44.7%) patients (Table 1). In total, 355 patients experienced Clavien-Dindo grade ≥II complications postoperatively, with an overall morbidity rate of 26.9%. Six deaths occurred during hospitalization, resulting in an in-hospital mortality rate of 0.5%. In addition, most patients had poor tumor differentiation, with 720 (54.5%) patients having moderate or low differentiation (Table 2). The pathological type of lung cancer was adenocarcinoma in 1,137 (86.0%) patients (Table 2).

**Table 2:** Characteristics of mSIS classification in surgical patients with NSCLC.

Characteristic	mSIS			*P-*value
	Score 0 (*N* = 819,62.0%)	Score 1 (*N* = 387,29.3%)	Score 2 (*N* = 115,8.7%)	
Age (years)	56(50, 63)	59(51, 67)	64(54, 70)	<0.001
Sex				<0.001
Male	282(34.4%)	191(49.4%)	76(66.1%)	
Female	537(65.7%)	196(50.6%)	39(33.9%)	
Smoking	162(19.8%)	122(31.5%)	54(47.0%)	<0.001
Drinking	139(17.0%)	59(15.2%)	16(13.9%)	0.966
Preoperative underlying diseases				0.367
No	465(56.8%)	204(52.7%)	61(53.0%)	
Yes	354(43.2%)	183(47.3%)	54(47.0%)	
Differential grade				0.037
Poor	99(12.1%)	63(16.3%)	23(20.0%)	
Middle	331(40.4%)	163(42.1%)	41(35.7%)	
Well	45(5.5%)	23(5.9%)	2(1.7%)	
Uncertain	344(42.0%)	138(35.7%)	49(42.6%)	
T stage				<0.001
T1a	387(47.3%)	138(35.7%)	30(26.1%)	
T1b	255(31.1%)	121(31.2%)	46(40.0%)	
T1c	121(14.8%)	53(13.7%)	14(12.2%)	
≥T2	56(6.8%)	75(19.4%)	25(21.7%)	
*N* stage				0.98
N0	718(87.7%)	338(87.3%)	101(87.8%)	
N1–3	101(12.3%)	49(12.7%)	14(12.2%)	
Histological subtypes				<0.001
Adenocarcinoma	750(91.6%)	314(81.1%)	73(63.5%)	
Squamous cell carcinoma	36(4.4%)	53(13.7%)	25(21.7%)	
Others	33(4.0%)	20(5.2%)	17(14.8%)	

**Figure 1:**
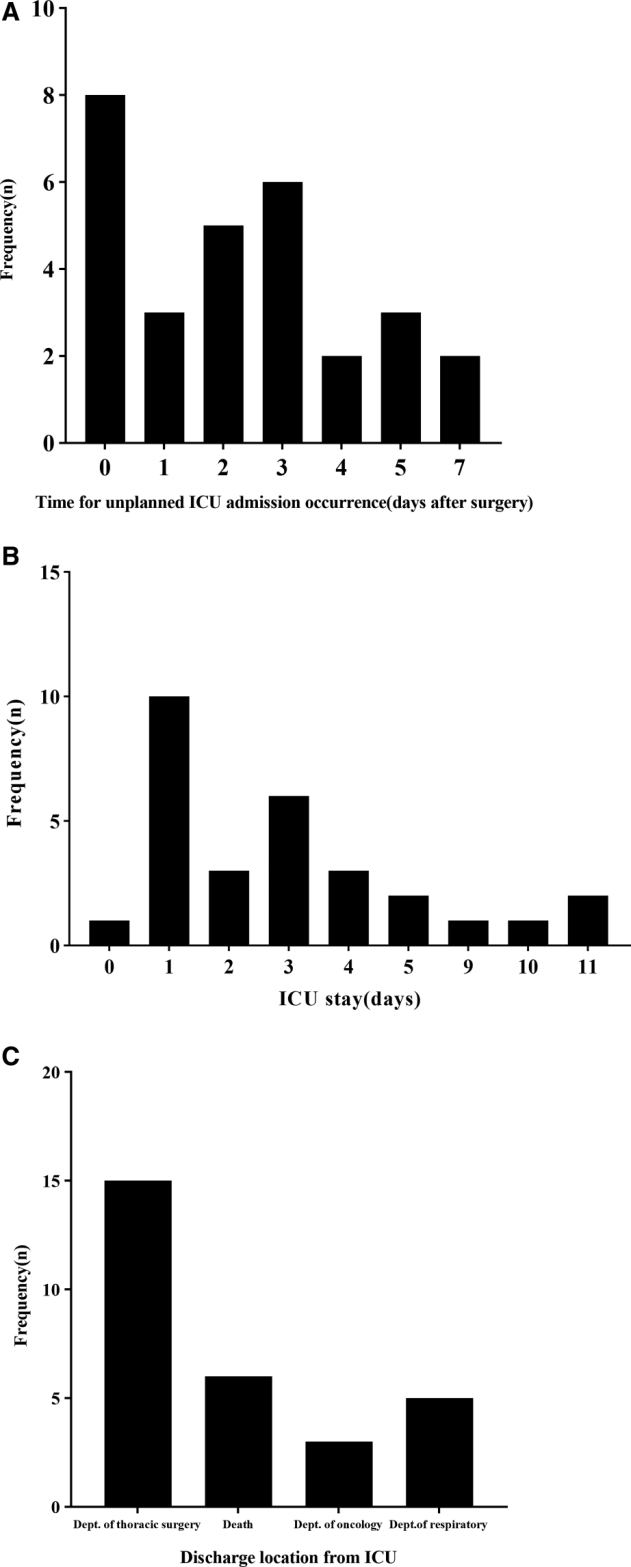
Characteristics of patients who underwent unplanned ICU admission. (**A**) Time of unplanned ICU admission occurrence after surgery. (**B**) Length of ICU stay after unplanned ICU admission. (**C**) Destinations of patients after discharge from the ICU.

There were 29 (2.19%) patients with unplanned ICU admissions, including 21 (72.4%) males and 8 (27.6%) females. The median age was 66 years (range 46–78 years), The main cause of unplanned admission to the ICU was respiratory failure (18 cases, 62.1%) (Table 3). The median number of postoperative days before unplanned ICU admission was 2 (0 d, 3.5 d) (Figure 1A). The median number of days of unplanned ICU stay was 3 (1 d, 4 d) (Figure 1B). After a stay in the ICU, the patients were primarily transferred to the thoracic surgery, respiratory or neurology ward, however 6 (20.7%) patients died (Figure 1C). In addition, the median duration of thoracic drainage tube placement in unplanned ICU patients was 6 d (4 d, 10.5 d), and the median total hospital stay was 23 d (15 d, 28.5 d) (Table 1).

**Table 3:** Reasons for postoperative unplanned ICU admission.

Classification^a^	*N*	%
Respiratory failure	18	62.1%
Cerebral infarction	4	13.8%
Cerebral hemorrhage	2	6.9%
Acute heart failure	1	3.4%
Severe renal failure	1	3.4%
Arrhythmia	2	6.9%
Pulmonary embolism	1	3.4%

^a^ Unplanned admission to the ICU could be due to two or more reasons.

### Relationship between the mSIS and clinicopathological features of patients with NSCLC

We analyzed the characteristics of the mSIS classification in patients with NSCLC. The results showed that 819 patients (62.0%) had a score of 0, 387 patients (29.3%) had a score of 1, and 115 patients (8.7%) had a score of 2. The results showed that compared with patients with mSIS = 0 and mSIS = 1, the patients with mSIS = 2 were older (*P* < 0.001), were more likely to be male (*P* < 0.001), have poorly differentiated tumors (*P* < 0.037), and have a later T stage (*P* < 0.001), had a higher smoking rate (*P* < 0.001) and had a higher proportion of squamous cell carcinoma (*P* < 0.001) (Table 2).

### Univariate and multivariate analyses of influencing factors

**Figure 2:**
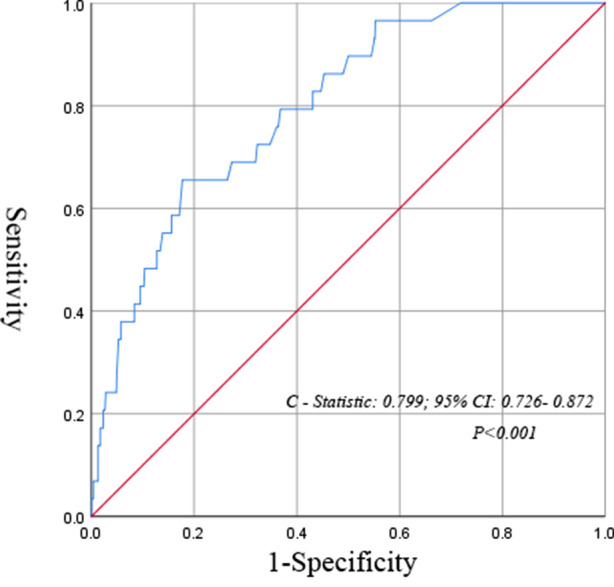
C-statistic revealing the discriminative power of the multivariable logistic regression model for prediction of postoperative unplanned ICU admission.

Univariate analysis showed that higher age (*P* < 0.001), higher mSIS score (*P* < 0.001), larger intraoperative infusion volume (*P* = 0.001), longer operation time (*P* = 0.002), male (*P* = 0.001), smoking history (*P* = 0.007), drinking history (*P* = 0.001) and preoperative underlying diseases (*P* = 0.002) were risk factors for unplanned admission to the ICU in postoperative patients with NSCLC. The results of the multivariate analysis showed that a mSIS of 2 (OR = 3.728; *P* = 0.004; 95% CI, 1.520–9.143), an alcohol consumption history (OR = 2.791, *P* = 0.011; 95% CI, 1.262–6.171), intraoperative infusion volume (OR = 1.001, *P* = 0.021; 95% CI, 1.000–1.001) and preoperative underlying diseases (OR = 3. 57, *P* = 0.004; 95% CI, 1.497–8.552) were independent risk factors for unplanned admission to the ICU after lung cancer surgery (Table 4). The multivariate logistic regression model showed that the C-statistic value was 0.799 (95% CI, 0.726∼0.872, *P* < 0.001) (Figure 2).

**Table 4:** Univariate and multivariable analysis of patients with unplanned ICU admission.

Characteristic	Univariate			Multivariable		
	OR	*P*-value	95% CI	OR	*P*-value	95% CI
Age (years)	1.088	<0.001	1.045∼1.131			
Sex	0.263	0.001	0.116∼0.599			
mSIS						
0	1			1		
1	0.906	0.84	0.345∼2.375	0.769	0.60	0.289∼2.051
2	4.882	<0.001	2.063∼11.555	3.728	0.004	1.520∼9.143
Drinking	3.809	0.001	1.792∼8.097	2.791	0.01	1.262∼6.171
Smoking	2.788	0.007	1.332∼5.839			
Operation time	1.009	0.002	1.004∼1.015			
Intraoperative infusion volume	1.001	0.001	1.000∼1.001	1.001	0.02	1.000∼1.001
Preoperative Underlying diseases	3.993	0.002	1.694∼9.414	3.578	0.004	1.497∼8.552

## Discussion

In this study, we analyzed the influencing factors of unplanned admission to the ICU in 1,321 patients with NSCLC after surgery. We found that a mSIS of 2 (ALB < 4.0 g/d and LMR < 3.4), an alcohol consumption history, a large amount of intraoperative infusion and preoperative underlying diseases were independent risk factors for unplanned admission to the ICU in patients with NSCLC after surgery, providing a good basis for the prediction of short-term adverse risk in patients with NSCLC. In addition, we also found that the mSIS was significantly correlated with tumor differentiation, T stage and tumor pathological type.

The mSIS is based on routine clinical laboratory indicators, including serum ALB, lymphocyte counts and monocyte counts, which makes it inexpensive and easy to use. The score reflects not only the level of inflammation but also the nutritional status of the patient (15). Chang (16) first developed the systemic inflammation score (SIS) to predict the postoperative prognosis of patients with renal clear cell carcinoma. Thereafter, Li Shuang Jiang (17) applied the systemic inflammation score (SIS) to lung cancer for the first time, and analyzed the prognosis of 390 patients with NSCLC. Their research team found that higher systemic inflammation score (SIS) scores were associated with lower rates of overall survival (OS) and disease-free survival (DFS).

The SIS is widely used in esophageal cancer (18–20), gastric cancer (21, 22), thyroid cancer (23), breast cancer (24), and liver cancer (25). Previous studies have found that the SIS plays an important role in predicting the prognosis of cancer patients after surgery. The mSIS was modified from the SIS to include a fixed value of 3.4 for the LMR as opposed to the median. Recently, more scholars have adopted the mSIS (7, 8, 23, 26). We found that the mSIS was significantly correlated with postoperative unplanned admission to the ICU in patients with NSCLC, especially in patients with a mSIS of 2 (ALB < 4.0 g/d and LMR < 3.4). This may be related to lower circulating protein levels, lower lymphocyte counts and higher monocyte counts in these patients. Serum ALB can be used to reflect not only the nutritional status of patients but also persistent systemic inflammatory responses (27). Therefore, patients with lower serum ALB levels may have a lower tolerance to surgery, resulting in an increased risk of short-term postoperative adverse events. In addition, the decrease in the lymphocyte count leads to a decrease in the immune response, affecting the monitoring and inhibition of tumor cells, which may lead to a poor prognosis (17). Previous studies have reported that circulating monocytes may promote cancer growth and metastasis of tumor cells while reducing immune surveillance (28). Our study found that the smoking status of patients influenced the mSIS, and smoking patients tended to have higher scores, which may have occurred because smoking leads to a sustained systemic inflammatory response (29). In addition, patients with mSIS = 2 had a higher proportion of squamous carcinomas and a lower proportion of poorly differentiated and more advanced T stages, which is similar to the results of previous studies (17, 30). This may be related to the presence of an immunosuppressive state in patients.

Previous studies have shown that lymphocytes have a key role in anti-cancer immunity (31). Circulating monocytes in the cancer microenvironment differentiate into macrophages (32). However, tumor-associated macrophages can promote angiogenesis, tumor cell growth, invasion and metastasis (33). mSIS is defined by three parameters, albumin level, lymphocyte count, and monocyte count. Therefore, a higher mSIS indicates that the patient is immunosuppressed (8), so the combination of factors indicate a higher risk of ICU admission. An increasing number of studies have shown that inflammation plays an important role in the development of deep venous thrombosis (DVT), which may be related to a variety of common cytokines involved in these two processes (34). Inflammation can reduce the expression of endothelial anticoagulant factor through thrombomodulin and heparin proteoglycan, and increase the expression of procoagulant factor through leukocyte adhesion molecule (35). Lymphocytes and monocytes can release platelet-derived microparticles that interact with coagulation factors and play a role in coagulation cascades (36). Inflammation can promote the development of a thrombus by affecting the fibrinolytic system and inhibiting fibrinolysis (37). In addition, studies have shown that there is a significant correlation between LMR and the development of postoperative deep venous thrombosis (DVT) (38). The mSIS consists of LMR and ALB, and patients with high scores have chronic inflammatory responses, indicating that patients with a high mSIS may have a risk of thrombosis, which may increase the risk of unplanned admission to ICU. This suggests that the mSIS is of great value in predicting short-term adverse risks in patients after surgery. The mSIS status can be used as a marker to identify unplanned admissions to the ICU in patients with NSCLC after surgery.

We considered the neutrophil-lymphocyte ratio (NLR), platelet-lymphocyte ratio (PLR), prognostic nutritional index (PNI) and other inflammatory indexes which have been shown to be related to the prognosis of lung cancer in previous studies (39–42). However, in our study, these indexes were not significantly correlated with postoperative unplanned admission to the ICU in patients with NSCLC. This shows that the mSIS score is better than other inflammatory indexes in predicting the short-term adverse risk of NSCLC, which may be related to the fact that mSIS can reflect the nutritional status of patients. Our study suggests that improved preoperative nutritional and immune status may reduce the risk of unplanned ICU admission for patients. Therefore, the nutritional and immune status of patients can be improved by calculating the mSIS before surgery to reduce the risk of complications and unplanned ICU admission.

In this study, we also found that previous alcohol consumption, a large amount of intraoperative infusion and preoperative underlying diseases were independent risk factors for postoperative unplanned admission to the ICU in patients with NSCLC. Although previous studies have shown that previous alcohol consumption has no significant correlation with the occurrence and development of tumors and the prognosis of cancer patients, some studies have found that alcohol has an effect on postoperative awakening and early cognition in patients undergoing general anesthesia, which increases the risk of unplanned admission to the ICU after general anesthesia. Excessive intraoperative infusion increases the risk of hypothermia in patients (43), circulating blood volume, cardiac load and the risk of pulmonary infection. Several studies have shown that preoperative essential organ diseases are high-risk factors for perioperative complications in patients with lung cancer. Preoperative underlying diseases, especially heart and lung diseases (44), can lead to a decrease in the reserve capacity of a patient's organs (45), resulting in a decrease in the patient's tolerance to surgery and a decrease in the ability to recover from surgical trauma, which increases the risk of unplanned admission to the ICU after surgery.

We found that patients with NSCLC who did not plan to stay in the ICU had longer hospital stays and higher hospitalization costs, and such patients had significantly longer thoracic drainage times than patients without ICU admission. These results are similar to the results of previous studies. This further indicates that it is meaningful to predict the influencing factors of unplanned admission to the ICU in patients with NSCLC after surgery.

### Limitations

Our study was a single-center retrospective study and lacks validation with external data. Further prospective studies are needed to better control for potential confounding factors, including patient characteristics, for validation.

## Conclusions

The mSIS can be used to predict unplanned admission to the ICU in patients with NSCLC after surgery. In addition, previous alcohol consumption, a large amount of intraoperative infusion and preoperative underlying diseases were independent risk factors for postoperative unplanned admission to the ICU in patients with NSCLC.

## Data Availability

The raw data supporting the conclusions of this article will be made available by the authors, without undue reservation.
